# Prevalence and correlates of cognitive impairment in kidney transplant recipients

**DOI:** 10.1186/s12882-017-0570-1

**Published:** 2017-05-12

**Authors:** Aditi Gupta, Jonathan D. Mahnken, David K. Johnson, Tashra S. Thomas, Dipti Subramaniam, Tyler Polshak, Imran Gani, G. John Chen, Jeffrey M. Burns, Mark J. Sarnak

**Affiliations:** 1 Division of Nephrology, University of Kansas Medical Center, Kansas City, KS USA; 2 Department of Biostatistics, University of Kansas Medical Center, Kansas City, KS USA; 3 Department of Psychology, Alzheimer’s Disease Center, University of Kansas, Lawrence, KS USA; 4 Department of Internal Medicine, University of Kansas Medical Center, Kansas City, KS USA; 5 Division of Internal Medicine, University of Kansas Medical Center, Kansas City, KS USA; 6 Department of Neurology, Alzheimer’s Disease Center, University of Kansas Medical Center, Kansas City, KS USA; 7 Division of Nephrology, Tufts Medical Center, Boston, MA USA; 8 Division of Nephrology and Hypertension, Department of Medicine, University of Kansas Medical Center, 3901 Rainbow Blvd, Kansas City, KS 66160 USA

**Keywords:** Cognitive impairment, Kidney transplantation, MoCA

## Abstract

**Background:**

There is a high prevalence of cognitive impairment in dialysis patients. The prevalence of cognitive impairment after kidney transplantation is unknown.

**Methods:**

*Study Design*: Cross-sectional study*. Setting and Participants*: Single center study of prevalent kidney transplant recipients from a transplant clinic in a large academic center. *Intervention*: Assessment of cognition using the Montreal Cognitive Assessment (MoCA). Demographic and clinical variables associated with cognitive impairment were also examined. *Outcomes and Measurements*: a) Prevalence of cognitive impairment defined by a MoCA score of <26. b) Multivariable linear and logistic regression to examine the association of demographic and clinical factors with cognitive impairment.

**Results:**

Data from 226 patients were analyzed. Mean (SD) age was 54 (13.4) years, 73% were white, 60% were male, 37% had diabetes, 58% had an education level of college or above, and the mean (SD) time since kidney transplant was 3.4 (4.1) years. The prevalence of cognitive impairment was 58.0%. Multivariable linear regression demonstrated that older age, male gender and absence of diabetes were associated with lower MoCA scores (*p* < 0.01 for all). Estimated glomerular filtration rate (eGFR) was not associated with level of cognition. The logistic regression analysis confirmed the association of older age with cognitive impairment.

**Conclusion:**

Cognitive impairment is common in prevalent kidney transplant recipients, at a younger age compared to general population, and is associated with certain demographic variables, but not level of eGFR.

**Electronic supplementary material:**

The online version of this article (doi:10.1186/s12882-017-0570-1) contains supplementary material, which is available to authorized users.

## Background

Cognitive impairment is prevalent in as many as 50 to 87% of dialysis patients [1, 2] and influences quality of life, employment rates, treatment adherence, hospital admissions, health care costs, morbidity and mortality [3–5]. Kidney transplant recipients, like patients on dialysis, have several risk factors for cognitive impairment such as comorbid illness, depression, and lower level of physical activity [6, 7]. Kidney transplantation offers quality of life and survival advantages over dialysis [8]. Cognitive impairment in kidney transplant recipients is associated with increased mortality [9, 10]. Some studies suggest cognitive impairment, especially in domains of verbal learning, memory and executive functioning in kidney transplant recipients when compared to healthy controls [11]. However, the actual prevalence of cognitive impairment in transplant recipients is unknown.

The knowledge of the prevalence of cognitive impairment is an important first step for designing future studies to assess the clinical impact of cognitive impairment and develop management strategies. Some studies indicate that cognition improves with kidney transplantation [12–16]. These studies, however, do not assess the prevalence of cognitive impairment in kidney transplant recipients as they were designed specifically to evaluate the impact of transplantation on cognition. The aim of this study is to evaluate the prevalence of cognitive impairment in kidney transplant recipients by screening eligible kidney transplant recipients. In addition, we evaluated the factors associated with cognitive impairment.

## Methods

### Study population

Adult kidney transplant recipients followed at the University of Kansas Kidney Transplant Clinic were included if they were i) English speaking; ii) without a history of acute stroke, concussion or traumatic brain injury within 2 months of testing and iii) with no acute illness at the time of participation. Patients were excluded if they i) had hearing or visual impairment; ii) were unable to read, write, speak or understand English; iii) had uncontrolled psychosis or seizure disorder; iv) were currently using antipsychotics or anti-epileptics v) received dual organ transplantation or vi) had a known history of cognitive impairment or dementia. Cognition was assessed during the clinic visit for post-transplant care. Eligible patients who attended the transplant clinic between May 2015 and June 2016 were approached. To minimize the acute effect of high dose steroids, surgery, and possible post-operative complications, cognitive assessments were performed only after the patient was at least 1 month post-transplant. As part of a standardized protocol, all patients were maintained on a mycophenolic acid compound and a calcineurin inhibitor with or without prednisone.

### Outcome variable

Cognition was assessed with the Montreal Cognitive Assessment (MoCA), a brief cognitive screening instrument (Additional file 1) [17]. The MoCA is a validated, clinic-based tool that samples from various domains of cognition and is sensitive in detecting mild cognitive impairment in several diseases, including Alzheimer’s disease and vascular dementia [18, 19]. We chose MoCA over the more commonly used mini-mental state exam (MMSE) for detecting cognitive impairment, as MoCA has more focus on executive function, a domain more commonly affected in kidney disease [20]. Moreover, MoCA has been successfully used in kidney disease, with a good correlation with detailed neuropsychological testing and better results than MMSE [21]. MoCA consists of a single page test with a maximum score of 30. The MoCA takes less than 10 min to complete and assesses seven domains of cognition: visuospatial/executive, naming, memory (delayed recall), attention, language, abstraction and orientation. The original English version 7.1 was used (http://www.mocatest.org/paper-tests/moca-test-full/). Based on published validation data on patients from a memory clinic, we defined cognitive impairment as MoCA score of less than 26 [17]. The MoCA was administered by medical assistants who underwent an hour of training that included detailed review of the online instructions along with practice sessions on mock patients. These medical assistants performed the test on the transplant recipients in a private examination room during the patients routine clinic visit to the transplant center.

### Exposure variables

Demographic and clinical variables that can potentially influence cognition such as age, gender, ethnicity, level of education, body mass index (BMI), blood pressure, history of smoking, diabetes, coronary artery disease, atrial fibrillation, transient ischemic attack (TIA) or stroke, cause for ESRD, serum hemoglobin, estimated glomerular filtration rate (eGFR), time on dialysis prior to transplant, and time after transplant were included in the analysis. Body mass index (BMI) and blood pressure were collected by medical assistants immediately before administration of MoCA. Demographic information and medical history were obtained through review of the patient’s electronic medical record. Diabetes was defined as current or past use of oral hypoglycemic agents or insulin. Coronary artery disease was defined as history of myocardial infarction, angina pectoris, angioplasty or coronary artery bypass surgery. Hypertension was defined by past or current use of antihypertensive agents. However, hypertension was not included in the final analysis as all patients were on one or more antihypertensive agent at some point in time. The eGFR calculated by the Modification of Diet in Renal Disease equation closest to the day of MOCA assessment (the same day in most cases) was collected.

The data collection was initiated as a quality improvement project and was approved by the Institutional Review Board. Data were collected on paper forms and managed in standard case report forms in REDCap (Research Electronic Data Capture) a web-based, electronic data capture tool hosted on a secure, password protected, HIPAA compliant server [22].

### Statistical analysis

We compared baseline covariate values between patients with and without cognitive impairment using the *t* test for continuous variables (or rank sum test when *t* test assumptions where violated as indicated by residual analysis) and the Pearson’s chi square test for categorical variables (or Fisher’s exact test when expected cell counts were insufficient for Pearson’s chi square test). To ensure accuracy of data collection (MoCA scores as well as data collected from patients medical records), two authors reexamined all the data entered for 50 random patients. We calculated the mean and the standard deviation for continuous variables, and the frequencies and the proportions for categorical variables. We further analyzed the prevalence in patients categorized by five-year age groups. With the MoCA score being a continuous variable, we performed a multivariable linear regression analysis to study the association of these variables with cognitive performance. In model 1 of the multivariable regression analysis, we included all variables of interest that can affect cognition (age, gender, race, level of education, history of diabetes, coronary artery disease, smoking, stroke, and atrial fibrillation, BMI, serum hemoglobin, estimated glomerular filtration rate, systolic and diastolic blood pressure, time of dialysis, time since kidney transplant, and end stage renal disease secondary to diabetes). We used the adjusted R-square to determine model 2, in which we included selected variables from model 1 on the basis of their predictive relevance. The adjusted R-square criterion assesses the improvement in the amount of variation described relative to the number of parameters in the model. Residual analyses were conducted to assess the normality and constant variance assumptions of the multivariable linear regression models. We also assessed the adjusted associations of the variables selected for Model 2 with cognitive impairment defined as a MoCA score < 26. All tests were two-tailed. For all analyses, *P* < 0.05 was considered statistically significant. All analyses were performed using SAS version 9.4 (SAS Institute Inc., Cary, NC, 2002–2012).

## Results

### Patient characteristics

A total of 297 patients were approached for the MoCA test. Out of these, 22 patients were excluded from the analysis due to known history of mental retardation (five), stroke causing known cognitive deficits (two), hearing impairment (three), visual impairment (five), or inability to speak fluent English (seven). Eight patients refused to complete the assessment and two started the assessment, but did not complete it. After excluding the above 32 patients, 265 kidney transplant recipients completed the MoCA. Of these, 226 (>85%) had complete data and were included in the analyses. Examination of data for 50 random patients (total of 1250 data points) by two authors revealed high accuracy of data with a total of seven errors. As shown in Table 1, the mean age of the participants was 54.0 (SD 13.4) years. Participants were predominantly white, male, and with an education level of college or above. About half of the study participants were obese defined by a BMI ≥ 30 (50.9%). The mean eGFR was 52 ± 21 ml/min/1.73 m^2^. The average time on dialysis prior to transplant was 2.3 years (SD 2.1) and the average time since transplant was 3.4 years (SD 4.1). Only 3% of the participants had a history of TIA or stroke and 21% had a history of coronary artery disease.

**Table 1:** Demographic and clinical characteristics of study participants

Patient characteristics	All	MoCA <26	MoCA ≥26	*p*-value
	*N* = 226	*N* = 131	*N* = 95	
Age (years)	54 (13.4)	56.7 (13.5)	51.3 (12.8)	<0.01
Gender				0.02
*Female*	89 (39.4)	43 (32.8)	46 (48.4)	
Race				0.57
*Caucasian*	165 (73.0)	93 (71.0)	72 (75.8)	
*African American*	43 (19.0)	28 (21.4)	15 (15.8)	
*Other race*	18 (8.0)	10 (7.6)	8 (8.4)	
Education level				0.08
*≥ College*	130 (57.5)	69 (52.7)	61 (64.2)	
BMI				0.65
*≥ 30 (kg/m* ^*2*^ *)*	115 (50.9)	65 (50)	50 (52.6)	
Blood Pressure				
*Systolic (mmHg)*	139 (18)	140 (19)	137 (17)	0.29
*Diastolic (mmHg)*	73 (12)	72 (12)	74 (13)	0.09
Serum hemoglobin (g/dl)	12.5 (2)	12.7 (2.1)	12.1 (1.9)	0.03
eGFR (ml/min/1.73 m^2^)	52 (21)	53 (21)	51 (22)	0.38
Time since KT (years)	3.4 (4.1)	3.3 (3.6)	3.6 (4.8)	0.72^a^
Time on dialysis (years)	2.3 (2.1)	2.3 (1.93)	2.2 (2.3)	0.16^a^
H/o smoking	95 (42.0)	57 (43.5)	38 (40.0)	0.60
H/o TIA/stroke	7 (3.1)	4 (3.1)	3 (3.2)	>0.99^a^
H/o diabetes	84 (37.2)	49 (37.4)	35 (36.8)	0.93
H/o CAD	42 (21.2)	26 (19.9)	16 (16.8)	0.57
H/o Atrial Fibrillation	25 (11.1)	16 (12.2)	9 (9.5)	0.52
ESRD secondary to diabetes	57 (25.2)	34 (26.0)	23 (24.2)	0.77

*Continuous variables = Mean (Standard deviation), Categorical variables = N (%)*

The *p*-values indicate the comparison between MoCA <26 and MoCA ≥26.

*BMI* body mass index, *eGFR* estimated glomerular filtration rate, *KT* kidney transplant, *H/o* history of, *TIA* transient ischemic attack, *CAD* coronary artery disease

^a^ *Nonparametric test*

### Cognitive assessment

Fifty eight percent reached criteria for cognitive impairment. Table 1 compares the patients with and without cognitive impairment. The mean age of patients with cognitive impairment was higher than patients without cognitive impairment (*p* ≤ 0.01), and a lower proportion of female patients had cognitive impairment (*p* = 0.02). Race, level of education, BMI, systolic and diastolic blood pressure, eGFR, time since transplantation, time on dialysis prior to transplantation, and a history of smoking, coronary artery disease, atrial fibrillation, diabetes, or the cause of ESRD were not different among those with and without cognitive impairment.

In the bivariate analysis, higher MOCA scores were associated with lower age (*p* < 0.001), female gender (*p* < 0.001), lower serum hemoglobin levels (*p* = 0.015), and higher level of education (*p* = 0.003). There was no association of the MOCA score with race, BMI, blood pressure, eGFR, time since kidney transplantation, time on dialysis prior to transplantation, history of smoking, stroke, diabetes, coronary artery disease, atrial fibrillation, and ESRD secondary to diabetes. Multivariable linear regression demonstrated that higher MOCA score was associated with younger age, female gender, a higher level of education, and diabetic status (Table 2). Model 1 includes all variables of interest entered in the analysis. Model 2 includes variables selected on the basis of their predictive relevance based on the adjusted R-square (Additional file 2).

**Table 2:** Association of demographic and clinical variables with the level of cognition

Model 1			
Exposure variable	B Coefficient	95% Confidence interval	*p*-value
Age (/10 years)	−0.659	−1.04 to −0.28	<0.001
Female	1.321	0.41 to 2.23	0.01
African American^a^	−1.189	−2.31 to −0.05	0.04
Other race^a^	−0.122	−1.71 to 1.47	0.88
College education or above	0.826	−0.04 to 1.70	0.06
H/o diabetes	1.594	0.35 to 2.83	0.01
H/o CAD	−0.158	−1.33 to 1.01	0.79
H/o smoking	−0.573	−1.44 to 0.30	0.20
H/o TIA/stroke	0.381	−2.07 to 2.83	0.76
H/o A.fib	0.787	−0.65 to 2.22	0.28
BMI (≥30 kg/m^2^)	0.343	−0.52 to 1.20	0.43
Serum hemoglobin (per g/dl)	−0.137	−0.38 to 0.11	0.27
eGFR (per ml/min/1.73 m^2^)	−0.005	−0.03 to 0.02	0.62
SBP (per mm Hg)	−0.011	−0.04 to 0.02	0.44
DBP (per mm Hg)	0.042	0.001 to 0.08	0.05
Time on dialysis (per year)	−0.104	−0.31 to 0.11	0.33
Time since KT (per year)	0.005	−0.11 to 0.12	0.93
ESRD secondary to diabetes^b^	0.027	−1.33 to 1.38	0.97
Model 2			
Exposure Variable	B Coefficient	95% Confidence interval	*p*-value
Age (/10 years)	−0.669	−1.00 to −0.34	<0.001
Female	1.363	0.50 to 2.23	0.002
College education or above	0.922	0.09 to 1.76	0.03
H/o diabetes	1.455	0.56 to 2.35	0.002
H/o smoking	−0.491	−1.34 to 0.36	0.25
H/o A. fib	−0.759	−0.58 to 2.10	0.27
Serum hemoglobin (per g/dl)	−0.120	−0.33 to 0.09	0.27
DBP (per mm Hg)	0.026	−0.01 to 0.06	0.15
Time on dialysis (per year)	−0.000	−0.35 to 0.04	0.12

Model 1 includes all variables of interest entered in the multivariable analysis. All variables are adjusted for the other variables. Models 2 includes variables selected on the basis of their predictive relevance using adjusted R-square criterion

*H/o* history of, *ESRD* end stage renal disease, *TIA* transient ischemic attack, *BMI* body mass index, *eGFR* estimated glomerular filtration rate, *SBP* systolic blood pressure, *DBP* diastolic blood pressure, *KT* kidney transplant

^a^reference group: Caucasian race. ^b^reference group: other causes of ESRD

**Fig. 1:**
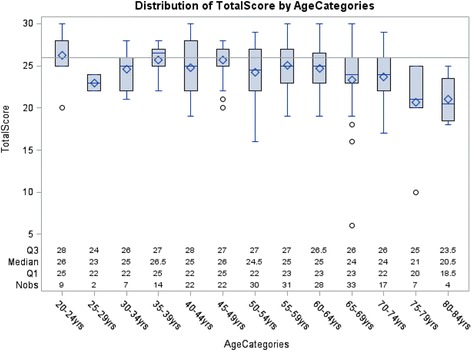
Boxplot of MoCA scores by age range

When considering the dichotomous outcome of cognitive impairment older age was associated with higher risk (Table 3). With the higher prevalence of cognitive impairment in older transplant recipients, we also analyzed the prevalence of cognitive impairment by five-year age groups (Fig. 1). These results demonstrate an increased prevalence of cognitive impairment with older age, but notably also a high prevalence of cognitive impairment even in individuals less than 50 years old.

**Table 3:** Determinants of cognitive impairment^a^

Exposure variable	Adjusted odds ratio	95% Confidence interval	*p*-value
Age (per 10 year increase)	1.33	1.06 to 1.69	0.02
Female	0.57	0.32 to 1.03	0.06
College education or above	0.73	0.41 to 1.30	0.28
H/o diabetes	0.75	0.40 to 1.39	0.37
H/o smoking	1.01	0.56 to 1.82	0.98
H/o A. fib	0.88	0.34 to 2.27	0.79
Serum hemoglobin (per g/dl)	1.11	0.96 to 1.28	0.59
DBP (per mm Hg)	0.98	0.96 to 1.01	0.21
Time on dialysis (per year)	1.04	0.91 to 1.19	0.59

^a^Association of clinical variables with a MOCA score < 26 using logistic regression

## Discussion

We examined the prevalence and covariates of cognitive impairment in a cohort of kidney transplant recipients. A majority of the transplant recipients with mean age of 54 years had cognitive impairment with the overall prevalence being more than two times the prevalence in those 65 years or older in the general population [23, 24]. Lower MoCA scores were associated with older age, male gender, and a lower level of education. These data underscore that cognitive impairment is common in kidney transplant recipients and additional studies are needed so as to understand the pathophysiology and impact of cognitive impairment in these individuals.

Several studies have shown that cognitive impairment is common in dialysis patients [1, 2]. In this study we show a high prevalence of cognitive impairment in kidney transplant recipients. We noted a higher prevalence of cognitive impairment with higher age. This trend is similar to that seen in the general population but occurs at a much younger age in kidney transplant recipients. For example, compared to a prevalence of 16–20% in adults 65 years or older in the general population [23, 24], we noted that a majority of the transplant recipients had cognitive impairment even at age of 40 years or less.

Not much is known about cognition and brain changes after kidney transplantation. Although cognition improves after kidney transplantation [12–16], our data suggest that that cognitive impairment in dialysis patients may not be entirely reversible. Several potential mechanisms may explain the cognitive impairment observed in kidney transplant recipients. Despite improvement in kidney function after transplantation, prolonged exposure to comorbid medical conditions including metabolic and vascular changes that are associated with kidney disease may result in non-reversible cerebrovascular disease that persists after successful transplantation. Alteration of the microbiome, immunomodulation, and neurotoxicity from medications such as calcineurin inhibitors or steroids may also contribute to cognitive impairment in transplant recipients.

We did not detect a relationship between cognition and eGFR. This is in contrast to pre-dialysis CKD where the severity of cognitive impairment is correlated with severity of renal dysfunction [4, 25]. This implies that the etiology of cognitive impairment in dialysis and kidney transplant recipients cannot be entirely attributed to a lower level of GFR but that other factors mentioned above contribute. Patients on dialysis are indeed exposed to metabolic and hemodynamic alterations that might contribute to cognitive impairment. Although there is an association with eGFR in pre-transplant CKD, cognitive impairment in this population may as well be due to several other factors such as vascular alterations, chronic inflammation and comorbid conditions such as diabetes and hypertension. It is also likely that cognitive impairment in pre-dialysis CKD takes several years to develop while level of eGFR in the transplant recipient only reflects a short period of the individual’s life-time exposure to cognitive risk.

Diabetes and hypertension have been associated with cognitive impairment and white matter hyperintensities in the brain [26, 27]. We did not note a relationship between the presence of diabetes and cognitive impairment. This may be due to selection bias in that the healthier diabetic patients receive kidney transplants.

Despite the similarity between the general population and the transplant recipients in age related cognitive decline, cognitive impairment in transplant recipients may be different from Alzheimer’s disease, the most common cause of cognitive impairment in the general population. For example, unlike Alzheimer’s disease [28], women performed better in the cognitive assessments than men. In addition, as alluded to above, the pathophysiology of cognitive impairment in kidney disease, and by extrapolation, in kidney transplantation recipients, more likely mimics vascular dementia rather than Alzheimer’s disease [20].

Our findings are clinically relevant. Cognitive impairment may have profound effects on outcomes of kidney transplantation [9, 10]. The care of transplant patients is a complex combination of polypharmacy, medication and dietary adherence, as well multiple outpatient clinic visits and tests. Medical adherence is affected by impairment in cognition and lack of adherence is a major cause for rejection and graft loss [29, 30]. Although mild cognitive impairment may not interfere notably with activities of daily living or be clinically evident, identifying these patients is important as cognitive impairment is a precursor to dementia with 5 to 20% of patients progressing to clinical dementia annually [31].

This study has some limitations. The cross sectional design precludes conclusions regarding cause and effect and does not provide information on whether cognition is stable, improving or deteriorating. Furthermore, it does not allow comparison of current level of cognition with that pre-transplant, or the association of current level of eGFR with decline in cognition in follow up. Although the prevalence of cognitive impairment was high, this may in fact be an underestimate as we excluded patients with known history of dementia. Depression can affect cognitive performance but was not assessed in this study. We excluded patients who did not speak English, which may affect generalizability. The clinical data were obtained from chart review which has its inherent limitations. We ensured accuracy of data collection by random sampling of 20% of the data points by two authors with 0.5% errors. In addition we used a structured case report form to abstract data. In this study we used MOCA to assess cognition. Although appropriate for this initial study, a screening tool such as MoCA may lack the specificity that can be achieved with more rigorous testing with detailed neurophysiological tests. However, for clinical practice, a simple tool such as MOCA is more practical. Furthermore, it is validated for use in outpatient clinics and can readily be applied to clinical practice in transplant clinics.

## Conclusions

The prevalence of cognitive impairment in kidney transplant recipients is high. In contrast to the general population even younger transplant recipients have a high prevalence of cognitive impairment. This information should be taken into consideration during patient education and monitoring of medical adherence. Further research is needed to understand the pathophysiology and consequences of cognitive impairment in transplant recipients. Strategies to help kidney transplant recipients cope with cognitive deficits should be developed.

## Additional files

Additional file 1: Description of the Montreal Cognitive Assessment. (DOCX 12 kb)

Additional file 2: Construction of model 2 in Table 2. (DOCX 11 kb)

## Abbreviations

BMI: Body mass index
CKD: Chronic kidney disease
eGFR: Estimated glomerular filtration rate
ESRD: End-stage renal disease
MoCA: Montreal Cognitive Assessment
TIA: Transient ischemic attack
