# Supplementary material for: Prevalence and correlates of cognitive impairment in kidney transplant recipients
Source: BMC Nephrol. 2017 May 12;18:158. doi: 10.1186/s12882-017-0570-1 (PMC5429555; doi:10.1186/s12882-017-0570-1)
Supplement: Supplementary file 1 — Description of the Montreal Cognitive Assessment. (DOCX 12 kb) [file 12882_2017_570_MOESM1_ESM.docx]

**Additional file 1**

**Description of the Montreal Cognitive Assessment**

The original English version 7.1 was used (http://www.mocatest.org/paper-tests/moca-test-full/). The medical assistants performed the test after undergoing an hour of training that included detailed review of the online instructions and practice sessions.

The MoCA is a single page test with a maximum score of 30. One point is added if the subject has ≤ 12 years of education. The MoCA takes less than 10 minutes to complete. It assesses seven domains of cognition: visuospatial/executive, naming, memory (delayed recall), attention, language, abstraction and orientation. The clock drawing test (three points) and a three dimensional cube drawing test (three points) test visuospatial cognitive abilities. An adapted version of the Trail Making B task (one point), a phonemic fluency task (one point), and a two-item verbal abstraction task (two points) test assess different aspects of the executive function. A sustained attention task consisting of target detection using tapping (one point), a serial subtraction task (three points), and digits forward and backward (one point each) assess attention, concentration, and working memory. A three-item confrontation naming task with low-familiarity animals (lion, camel, rhinoceros) (three points), repetition of two syntactically complex sentences (two points), and the aforementioned fluency task evaluate language. Two learning trials of five nouns and their delayed recall after approximately five minutes (five points) test delayed recall. The remaining six points are for orientation to time and place.
