# Supplementary material for: Prevalence and correlates of cognitive impairment in kidney transplant recipients
Source: BMC Nephrol. 2017 May 12;18:158. doi: 10.1186/s12882-017-0570-1 (PMC5429555; doi:10.1186/s12882-017-0570-1)
Supplement: Supplementary file 2 — Construction of model 2 in Table 2. (DOCX 11 kb) [file 12882_2017_570_MOESM2_ESM.docx]

**Additional file 2**

Construction of model 2 in table 2

For model 2 we removed variables in the following order with the adjusted R-squared in parenthesis: race (0.1480); ESRD secondary to diabetes (0.1520); stroke (0.1560); coronary artery disease (0.1598); time to kidney transplant (0.1636); eGFR (0.1665); BMI (0.1683); and blood pressure (0.1692). The remaining variables, i.e. age, gender, level of education, history of diabetes, history of smoking, history of atrial fibrillation, serum hemoglobin, diastolic blood pressure and time on dialysis prior to transplant were included in model 2.
